# Retraction: Advanced machine learning-guided optimization platform for high-yield soluble expression of *Pseudomonas aeruginosa* exotoxin A in engineered *Escherichia coli* strains

**DOI:** 10.1371/journal.pone.0353537

**Published:** 2026-07-13

**Authors:** The PLOS One Editors

The *PLOS One* Editors retract this article [1] due to concerns about:

- Compliance with PLOS policies on Authorship and Artificial Intelligence Tools and Technologies.
- Citation of unretrievable References 2, 3, 6, 7, 8, 18, 20, and 26.
- Citation of references that appear to have errors, References 11, 53, and 56.
- Citation of references that appear to be irrelevant in the cited context, References 25, 36, 39, 41, 42, 44, 54, and 55.

Before these concerns were shared with the authors, the first author requested correction of multiple reference citation errors, and had subsequently requested retraction. The second author had also requested retraction and informed PLOS that they are no longer affiliated with Shanghai Jiao Tong University and listed this affiliation on this article without authorization, and that the work presented in this article was not performed at this institution.

All authors either did not respond directly or could not be reached.
